# Model-Informed Precision Dosing of Linezolid in Patients with Drug-Resistant Tuberculosis

**DOI:** 10.3390/pharmaceutics14040753

**Published:** 2022-03-30

**Authors:** Laurynas Mockeliunas, Lina Keutzer, Marieke G. G. Sturkenboom, Mathieu S. Bolhuis, Lotte M. G. Hulskotte, Onno W. Akkerman, Ulrika S. H. Simonsson

**Affiliations:** 1 Department of Pharmaceutical Biosciences, Uppsala University, 75124 Uppsala, Sweden; laurynas.mockeliunas@farmbio.uu.se (L.M.); lina.keutzer@farmbio.uu.se (L.K.); 2 Department of Clinical Pharmacy and Pharmacology, University Medical Center Groningen, University of Groningen, 9713 GZ Groningen, The Netherlands; m.g.g.sturkenboom@umcg.nl (M.G.G.S.); m.s.bolhuis@umcg.nl (M.S.B.); l.m.g.hulskotte@student.rug.nl (L.M.G.H.); 3 Department of Pulmonary Diseases and Tuberculosis, University Medical Center Groningen, University of Groningen, 9713 GZ Groningen, The Netherlands; o.w.akkerman@umcg.nl; 4 Tuberculosis Center Beatrixoord, University Medical Center Groningen, University of Groningen, 9751 ND Groningen, The Netherlands

**Keywords:** tuberculosis, population pharmacokinetics, linezolid, auto-inhibition of linezolid elimination, model-informed precision dosing, simulation

## Abstract

Linezolid is an efficacious medication for the treatment of drug-resistant tuberculosis but has been associated with serious safety issues that can result in treatment interruption. The objectives of this study were thus to build a population pharmacokinetic model and to use the developed model to establish a model-informed precision dosing (MIPD) algorithm enabling safe and efficacious dosing in patients with multidrug- and extensively drug-resistant tuberculosis. Routine hospital therapeutic drug monitoring data, collected from 70 tuberculosis patients receiving linezolid, was used for model development. Efficacy and safety targets for MIPD were the ratio of unbound area under the concentration versus time curve between 0 and 24 h over minimal inhibitory concentration (*f*AUC_0–24h_/MIC) above 119 and unbound plasma trough concentration (*f*C_min_) below 1.38 mg/L, respectively. Model building was performed in NONMEM 7.4.3. The final population pharmacokinetic model consisted of a one-compartment model with transit absorption and concentration- and time-dependent auto-inhibition of elimination. A flat dose of 600 mg once daily was appropriate in 67.2% of the simulated patients from an efficacy and safety perspective. Using the here developed MIPD algorithm, the proportion of patients reaching the efficacy and safety target increased to 81.5% and 88.2% using information from two and three pharmacokinetic sampling occasions, respectively. This work proposes an MIPD approach for linezolid and suggests using three sampling occasions to derive an individualized dose that results in adequate efficacy and fewer safety concerns compared to flat dosing.

## 1. Introduction

Rifampicin-resistant (including multidrug-resistant (MDR)) tuberculosis (TB) is still a global health threat, with close to half a million new cases annually [1]. MDR-TB is defined as resistant to both isoniazid and rifampicin and extensively drug-resistant (XDR) as resistant to isoniazid and rifampicin, plus any fluoroquinolone and at least one Group A drug (levofloxacin, moxifloxacin, bedaquiline, or linezolid). Treatment of these infections requires the use of second-line treatment, which is longer, associated with higher costs, increased toxicity, and has a success rate of merely 59% [2,3]. Currently, one of the core second-line anti-TB drugs used for the treatment of MDR- and XDR-TB is linezolid, a synthetic antibiotic from the oxazolidinone class, inhibiting the bacterial protein synthesis by binding to the 23S rRNA of 50S ribosomal subunit [4]. Its two main inactive metabolites are hydroxyethyl glycine and aminoethoxy acetic acid, excreted both renally (unchanged) and non-renally [4]. The efficacy of linezolid has been suggested to be related to the ratio of unbound area under the concentration versus time curve between 0 and 24 h over minimal inhibitory concentration (*f*AUC_0–24h_/MIC) with a threshold above 119 [5,6]. As a potential safety target for linezolid in TB treatment, it has been suggested that the unbound plasma trough concentration (*f*C_min_) should be below 1.38 mg/L [6,7] since the time above *f*C_min_ is assumed to be related to mitochondrial toxicity [8,9].

Treatment of MDR- and XDR-TB with linezolid (nowadays usually in combination with one of the later generation fluoroquinolones, bedaquiline, and another second-line anti-TB drug) [10] is much longer than the standard treatment of other indications with linezolid that have a maximum treatment length of 28 days, which has been shown to lead to more serious adverse events [6,10]. A common adverse event, especially during longer treatment, is myelosuppression (mainly thrombocytopenia, but also leukopenia and anemia). Peripheral and optic neuropathy, lactic acidosis, hepatotoxicity, and hypoglycemia occur more seldom but can be severe and irreversible (neuropathies) [6,11]. Linezolid’s high toxicity during longer treatment contributes to a treatment discontinuation rate of 22.6% (141/624, based on 11 studies conducted between 2009 and 2018) [12]. One approach to reduce the risk of developing serious adverse events and minimize the risk of early treatment discontinuation is model-informed precision dosing (MIPD) [13]. MIPD is guided by patient characteristics, individual plasma drug concentrations, and a population pharmacokinetic (PK) or combined pharmacokinetic-pharmacodynamic (PKPD) model. The approach can be used in TB treatment to reduce the risk of treatment failure as well as toxicity [13,14,15,16]. The challenge in the treatment of MDR- and XDR-TB is to administer a linezolid dose that is highly efficacious with limited toxicity. MIPD can be used to support individual dose selection using a population PK model and targets for efficacy and safety.

The objectives of this work were to develop a population PK model, which, together with pre-set efficacy and safety targets, can be used to develop an MIPD algorithm enabling safe and efficacious dosing on an individual level.

## 2. Materials and Methods

### 2.1. Patients and Pharmacokinetic Data

Routine therapeutic drug monitoring (TDM) data from 70 MDR- or XDR-TB patients receiving linezolid was collected at the TB center Beatrixoord in Haren, University Medical Center Groningen (UMCG), The Netherlands, between 2007 and 2019. Due to the retrospective nature of this study and because TDM was already part of the routine treatment protocol in the TB center, the need for subjects to provide informed consent was waived by the Medical Ethical Review Board UMCG (METC 2013.492, ethical clearance date: 3 December 2013). Patient demographics, patient characteristics, linezolid total plasma concentrations, and linezolid dosing regimens were retrieved from the medical charts. A summary of patient demographics and characteristics is provided in Table 1. Linezolid was administered in combination with other anti-TB drugs for up to 542 days with oral daily doses (once daily (QD) or twice daily (BID)) ranging from 150 to 1200 mg. A summary of all regimens included in the analysis can be found in Table S1 (Supplementary Materials). Linezolid plasma concentrations were obtained at varying time points at up to seven independent sampling occasions in each patient. In most instances, a pre-dose sample was taken before drug administration. Plasma total linezolid concentrations were quantified using validated liquid chromatography coupled with the mass spectrometry (LC-MS/MS) (ThermoFisher, San Jose, CA, USA) method with a lower limit of quantification (LLOQ) of 0.05 mg/L [17].

### 2.2. Population Pharmacokinetic Model

A population pharmacokinetic model was developed based on data from 70 patients (811 observations). One individual’s second sampling occasion was excluded from the analysis as the treatment with linezolid was stopped one day before sampling. There were two observations below LLOQ, which were set to LLOQ/2 since the usage of likelihood-based methods such as the M3 and M4 method [19] did not seem necessary in light of the sparseness of LLOQ data.

Model comparison during the modeling process was performed by comparing the objective function value (OFV) of two nested hierarchical models, where a decrease in OFV of 3.84 for one degree of freedom (addition or removal of one parameter) is considered to be statistically significant at a 5% significance level according to the chi-squared distribution (χ^2^-distribution).

#### 2.2.1. Structural Model Building

Different disposition models were evaluated, including one- and two-compartment models. In order to describe absorption, a first-order absorption with and without lag-time and a transit absorption model [20,21] were tested. Transit absorption was hard-coded with an increasing number of transit compartments (NN) until the most optimal number of compartments was reached [20,21], as described in Equations (1) and (2). Equation (1) represents the first absorption transit compartment, while Equation (2) represents all other transit compartments.

(1) $$\frac{dA_{1}}{dt}=-k_{tr}\cdot A_{1}\;$$

(2) $$\frac{dA_{n}}{dt}=-k_{tr}\cdot A_{\left(n-1\right)}-k_{tr}\cdot A_{n}\;$$

*k_tr_* is the transit rate constant calculated as *k_tr_* = (NN + 1)/*MTT*, and *MTT* is the mean transit time (estimated). The amount of drug in a certain transit compartment is described by *A_n_*, where n is the absorption compartment.

For drug elimination, linear elimination, Michaelis–Menten elimination kinetics, as well as different approaches to account for drug-induced auto-inhibition of elimination [22,23,24] were explored. The first evaluated approach describing auto-inhibition of elimination was developed for linezolid by Plock et al. [22], where an empirical inhibition compartment is introduced, i.e., the drug concentration in the inhibition compartment drives the auto-inhibition. Different previously published rate constant into the inhibition compartment (*k_IC_*) and concentration in the inhibition compartment yielding half of clearance inhibition (*IC_50_*) values [22,25,26] were evaluated, and the ones providing the best fit were fixed and retained in the model. The second approach, initially developed for itraconazole [23], describes clearance inhibition dependent on dose with an exponential function. Lastly, in a model originally developed for auto-induction of rifampicin elimination [24], the formation of an enzyme is described by a first-order enzyme degradation and zero-order formation rate in which enzyme formation is stimulated by the presence of the drug via a nonlinear (E_max_) model. For description of linezolid elimination auto-inhibition, the approach was reversed by inhibiting the enzyme formation.

#### 2.2.2. Stochastic Model Building

Different residual error models on a normal scale were explored, including additive, proportional and combined additive plus proportional models. All possible combinations of inter-individual variability (IIV) and inter-occasion variability (IOV) were tested on all structural parameters. IIVs and IOVs were modeled exponentially, assuming that individual parameter values are log-normally distributed. Correlations were tested between IIVs of absorption parameters.

#### 2.2.3. Covariate Model Building

Allometric scaling of apparent clearance (*CL/F*) and apparent volume of distribution (*V/F*) was introduced using bodyweight as a descriptor for body size [27,28,29]. The exponents for the allometric relationships were fixed to 0.75 and 1 for *CL/F* and *V/F*, respectively [30], and the terms were scaled to 70 kg. The impact of additional covariates including age, sex, origin of birth (WHO region), HIV co-infection, diabetes, smoking, alcohol abuse, pre-emptive use of erythropoietin, creatinine clearance (calculated using the Cockcroft-Gault equation [18]) and the effects of concomitant P-glycoprotein (P-gp) inhibitors, P-gp inducers, CYP3A4 inhibitors, and CYP3A4 inducers were assessed using the automated stepwise covariate modeling (SCM) procedure in Perl-speaks-NONMEM (PsN) [31]. Values of calculated creatinine clearance above 150 mL/min were truncated to 150 mL/min. Only clinically plausible covariate relationships were explored (see Table S2, Supplementary Materials). Missing covariate information was handled by imputing the mean value of a covariate for continuous covariates and the mode for categorical covariates. Covariates were selected in a forward inclusion step at a statistical significance level of *p* < 0.05 and retained following a backward deletion step (*p* < 0.01). Statistically significant covariate relationships from the SCM were also assessed for clinical significance. Clinical significance was defined as a change in the typical parameter by more than 20% caused by the covariate effect for categorical covariates and 20% change from the median for the 10% and 90% percentiles of the continuous covariate. The covariates pregnancy, anti-retroviral therapy, and therapeutic use of erythropoietin were not evaluated since only 3, 4, and 0 patients, respectively, exhibited the particular covariate.

#### 2.2.4. Model Evaluation

Prediction-corrected visual predictive checks (pcVPCs), goodness-of-fit (GOF) plots, scientific plausibility, and precision of model parameter estimates were evaluated. A 1000 sample sampling importance resampling (SIR) procedure was performed in PsN for the final model to obtain the 90% nonparametric confidence interval for all parameters in order to assess parameter uncertainty.

### 2.3. Model-Informed Precision Dosing Algorithm

An MIPD algorithm originally developed for dose individualization of rifampicin [15] was adapted for dose optimization of linezolid treatment in patients with MDR- and XDR-TB.

A simulated population of 1000 hypothetical patients was created by bootstrapping patient covariates, as well as individual MIC values from the original study population (patient characteristics, see Table 1).

For the MIPD algorithm, the *f*AUC_0–24h_/MIC > 119 and *f*C_min_ < 1.38 mg/L were used as efficacy and safety targets [5,7], respectively, and the individualized dose should meet both the efficacy and the safety target.

In order to obtain observed linezolid plasma concentrations for the simulated patient population, the exposure following an initial dose of 600 mg QD was simulated for the first day of treatment. In the next step, these concentrations were used to compute individual PK parameters (empirical Bayes estimates (EBEs)), such as individual clearance or mean transit time. Based on the individual PK parameters, the individual *f*AUC_0–24h_/MIC and *f*C_min_ were derived following doses of 150 mg to 1200 mg QD and 150 mg to 600 mg BID (increments of 150 mg). The MIPD algorithm was then used to select the individual dose that meets the efficacy and safety target. In case both the efficacy and safety were reached, the lowest efficacious dose was selected. If two dosing regimens resulted in the same *f*AUC_0–24h_/MIC, which is the case for the same daily dose administered once versus twice daily, the dosing regimen leading to the lower *f*C_min_ was chosen, ensuring safety. If efficacy but not safety was reached, the lowest efficacious dose was selected, and a warning was given regarding safety. If safety but not efficacy was attained, the highest dose was chosen, and an efficacy warning was given. If neither efficacy nor safety could be achieved, the highest dose was selected (1200 mg QD), and warnings regarding efficacy and safety were reported. The selected individual dose was then used for simulation of further sampling occasions using an adaptive dosing strategy. Using this workflow, in total, three PK sampling occasions on days 1, 8, and 15 of treatment were simulated using a sparse sampling (0, 2, and 5 h post dose) [32], updating the individual PK parameters at every occasion using the newly obtained linezolid plasma concentrations.

For transformation of simulated total AUC_0–24h_ and C_min_ to *f*AUC_0–24h_ and *f*C_min_, respectively, linearity in protein binding across the simulated plasma concentration range was assumed, and AUC_0–24h_ and C_min_ were multiplied by the fraction unbound (assumed to be 0.69) [33].

In order to evaluate the performance of the MIPD algorithm, the true individual doses were derived using information from all sampling occasions and a rich PK sampling for EBE estimation.

To compare the performance of the MIPD algorithm using different amounts of information for computation of the individual PK parameters, the relative bias (*rBias*) Equation (3) and relative root mean squared error (*rRMSE*) Equation (4) were calculated for *f*AUC_0–24h_/MIC and *f*C_min_ as follows:

(3) $$rBias=\frac{1}{N}\sum_{1}^{i}\frac{\frac{predicted_{i}-observed_{i}}{predicted_{i}+observed_{i}}}{2}\times 100$$

(4) $$rRMSE=\sqrt{\frac{1}{N}\sum_{1}^{i}\frac{{\left(predicted_{i}-observed_{i}\right)}^{2}}{{\left(\frac{predicted_{i}+observed_{i}}{2}\right)}^{2}}\times 100\;}$$

The relative dose prediction error (*rDPE*), evaluating the accuracy in dose prediction, was computed as follows Equation (5):

(5) $$rDPE=\frac{predictedDD_{i}-trueDD_{i}}{trueDD_{i}}\times 100$$

where *DD* is the total daily dose.

### 2.4. Software

The data were analyzed with the non-linear mixed-effects modeling software NONMEM (v.7.4.3; Icon Development Solutions, Ellicott City, MD, USA) [34] using conditional estimation with interaction (FOCE-I). Data handling and visualization were performed in R (v.3.6.1; R Foundation for Statistical Computing, Vienna, Austria) [35]. Model diagnostics were generated using Xpose4 (v.4.6.1) [31] and prediction-corrected visual predictive checks (pcVPCs) were created with PsN (v.4.9.5) [31].

## 3. Results

### 3.1. Population Pharmacokinetic Model

The final population PK model consisted of a one-compartment disposition model since a two-compartment model did not describe the data statistically significantly better (*p* > 0.05). A transit absorption model including five transit compartments was statistically significantly superior to an absorption lag-time model. The addition of a sixth transit compartment did not improve the fit significantly. Incorporating Michaelis–Menten elimination kinetics did not improve the model fit (OFV: 1978.5) compared to a model with first-order elimination (OFV: 1974.9), and thus first-order kinetics were chosen for the description of linezolid elimination. Based on goodness-of-fit plots (GOF) plots and prediction-corrected visual predictive checks (pcVPCs), a slight underestimation at higher concentrations was observed, suggesting the need to explore concentration-dependent auto-inhibition of linezolid elimination. For that purpose, different models, including dose- and time-dependency [23] as well as concentration- and time-dependency [22,24], were tested to describe auto-inhibition of linezolid elimination. In the final model, the structure of a previously developed concentration- and time-dependent elimination model developed by Plock et al. [22] was implemented. The incorporated auto-inhibition model [22] consists of an empirical inhibition compartment. Depending on the concentration in the inhibition compartment (*C_i_*), clearance (*CL*) from the central compartment (*A_c_*) is inhibited, where *CL* is a fraction of the original uninhibited value at the first dose. Equations (6) and (7) describe the *CL* auto-inhibition:

(6) $$\frac{dA_{c}}{dt}=k_{a}\cdot A_{a}-\frac{CL}{V_{d}}\cdot A_{c}\cdot \left(RCLF+\left(1-RCLF\right)\cdot \left(1-\frac{C_{i}}{IC_{50}+C_{i}}\right)\right)\;$$

(7) $$\frac{dC_{i}}{dt}=k_{IC}\cdot \left(\frac{A_{c}}{V_{d}}-C_{i}\right)\;$$

where *CL* is the uninhibited clearance (*L*/h), *A_a_* the linezolid amount in the absorption compartment (mg), *A_c_* the linezolid amount in the central compartment (mg), *C_i_* the linezolid concentration in the inhibition compartment (mg/L), *k_a_* the absorption rate constant (h^−1^), *V_d_* the central volume of distribution (*L*), *k_IC_* the rate constant into the inhibition compartment (1/h), *RCLF* the remaining *CL* fraction, and *IC_50_* the concentration in the inhibition compartment leading to half of the maximum clearance inhibition (mg/L). The *k_IC_* was fixed to the best fitting literature value of 0.0005 h^−1^ [25] and *IC_50_* to 0.38 mg/L [25] due to the fact that most of the patient data in this study were captured in steady state, thus not enabling estimation of the inhibition parameters with sufficient precision.

The residual error model was a combined additive and proportional error on a normal scale. IIV in *CL/F* and mean transit time (*MTT*) were statistically significant, as well as IOV in *CL/F*, *V/F*, *MTT*, and *k_a_*. Covariances were not found to be statistically significant between any of the parameters.

The parameters *CL/F* and *V/F* were allometrically scaled using bodyweight. Out of all explored covariates, HIV on *CL/F*, sex on *k_a_*, administration of P-gp inhibitors on *MTT* were found to be both statistically and clinically significant.

**Figure 1:**
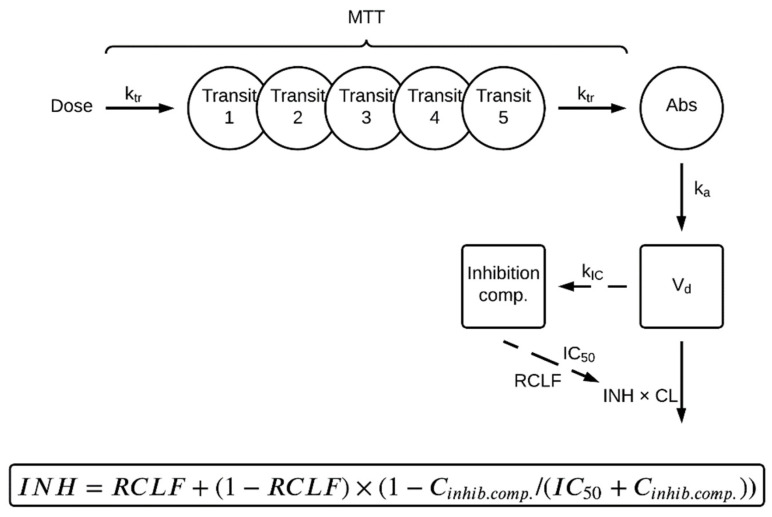
Schematic representation of the final linezolid population pharmacokinetic model. First, the dose is transferred into an absorption compartment (Abs) via five transit compartments (Transit 1–5), where *k_tr_* is the transit rate constant describing the transfer between transit compartments, calculated as the number of transit compartments (NN) + 1 divided by the mean transit time (*MTT*). The drug is absorbed from Abs to the central compartment (indicated by *V_d_*, the distribution volume of the central compartment), described by the absorption rate constant (*k_a_*). Clearance (*CL*) from the central compartment is inhibited based on the linezolid plasma concentration (*C_inhib.comp_*) in an empirical inhibition compartment (Inhibition comp.). The concentration- and time-dependency of the inhibition (*INH*) is described by the *C_inhib.comp_* leading to half of the maximum possible inhibition (*IC_50_*) and a rate constant (*k_IC_*) representing the transfer from the central into the inhibition compartment. The fraction of clearance remaining uninhibited is described by the parameter *RCLF*. The elimination of the drug is described by first-order kinetics, which is inhibited by *INH*.

**Figure 2:**
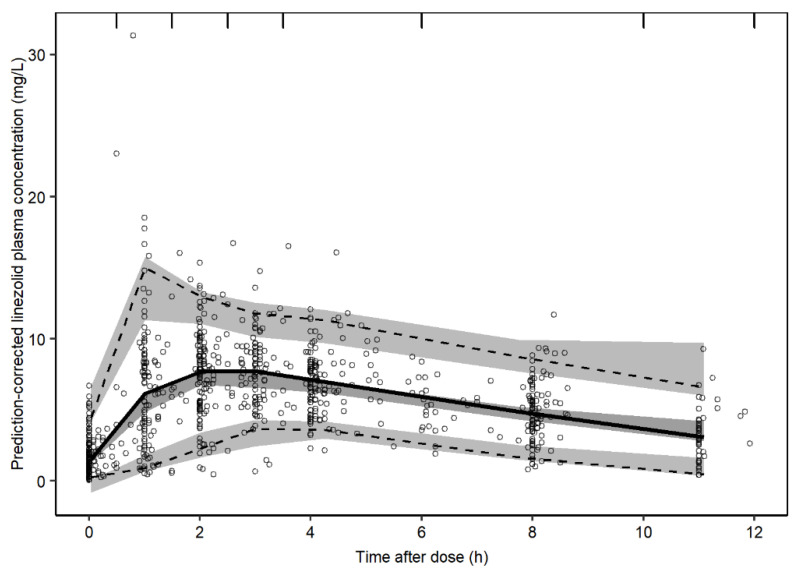
Prediction-corrected visual predictive check (pcVPC) of the final linezolid population pharmacokinetic model. The solid and dashed lines are the median, 2.5th and 97.5th percentiles of the observed data, respectively. The shaded areas (top to bottom) are the 95% confidence intervals of the 97.5th (light gray), median (gray), and 2.5th (light gray) percentiles of the simulated data based on 1000 simulations. Open circles are prediction-corrected observation points.

The NONMEM code for the final model is given in Text S1 (Supplementary Materials). Goodness-of-fit plots are shown in Figure S1 (Supplementary Materials). The structure of the final model is schematically represented in Figure 1, and the final parameter estimates are provided in Table 2. The final model described the observed data well in all dose groups based on the precision in parameter estimates, GOFs, individual plots (not shown), and pcVPCs showing both the whole population (Figure 2) as well as strata for the different patient covariates (Figure S2, Supplementary Materials).

### 3.2. MIPD Algorithm

**Figure 3:**
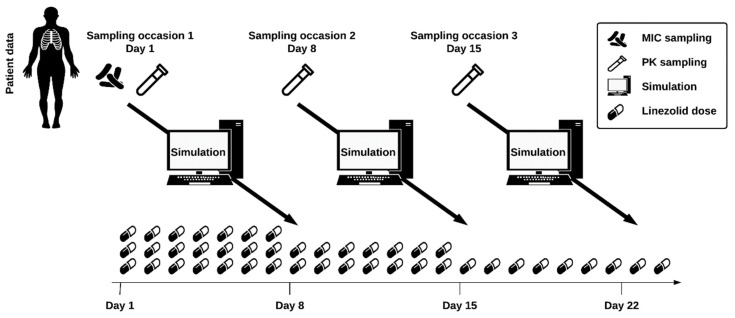
Model-informed precision dosing workflow. MIC determination is performed on day 1 of treatment and PK sampling on days 1, 8, and 15. A Bayesian forecast is then performed using the population PK model, patient characteristics, and individual linezolid plasma concentrations. Taking efficacy and safety into account, the dose is adjusted one week after PK sampling. MIC, minimal inhibitory concentration; PK, pharmacokinetics.

An MIPD algorithm incorporating adaptive dosing was developed for individualized linezolid dosing in patients with MDR- and XDR-TB. The proposed MIPD workflow is illustrated in Figure 3.

**Figure 4:**
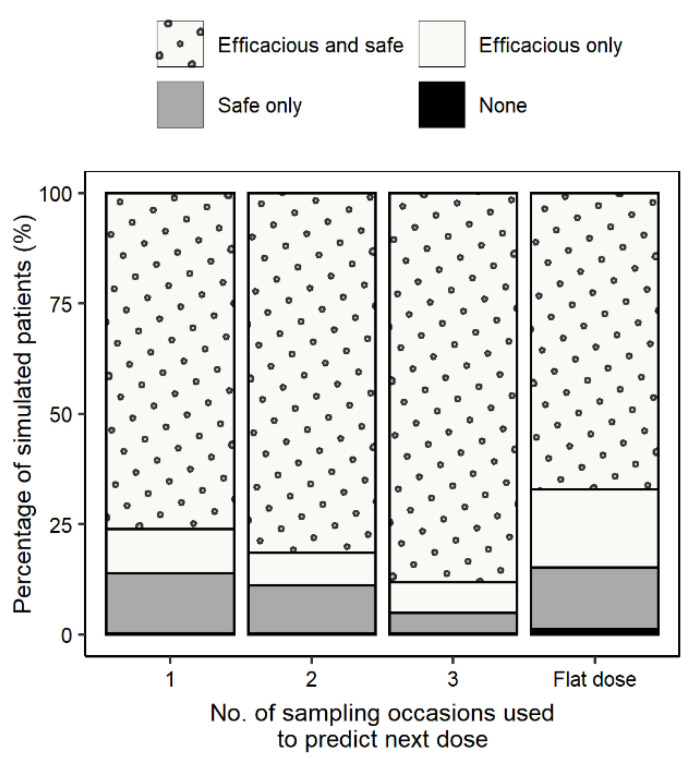
Percentage of patients reaching both efficacy and safety, only efficacy, only safety, and neither efficacy nor safety using information from one, two, and three sampling occasions to derive individual PK parameters. The true individual PK parameters were obtained from a rich sampling in order to derive the true optimal dose for comparison.

A flat dose of 600 mg QD led to efficacious and safe (*f*AUC_0–24h_/MIC > 119 and *f*C_min_ < 1.38 mg/L) exposures in 67.2% of the simulated patients (17.6% of the patients did not meet the safety, 14.0%, not the efficacy, and 1.2% neither the efficacy nor the safety target) (Figure 4). Using the MIPD approach, both the efficacy and safety targets were met in 76.1%, 81.5%, and 88.2% of the simulated patients following dosing regimens derived based on information from one, two, or three PK sampling occasions, respectively. Using information from three occasions, 6.9% of the simulated patients did not meet the safety target, 4.6% did not meet the efficacy target, and 0.3% did not meet the safety nor the efficacy target (Figure 4). A Sankey plot (Figure S3, Supplementary Materials) was created showing individual dose adjustments for three consecutive PK sampling occasions, indicating that a significant part of the simulated patients received the appropriate dose when adjusted based on information from the first sampling occasion. All doses selected by the MIPD algorithm were QD doses since BID dosing would lead to a higher *f*C_min_.

In order to determine how many sampling occasions are necessary to compute the individual PK parameters and subsequently predict the *f*AUC_0–24h_/MIC and *f*C_min_ with sufficient accuracy and precision, the *rRMSE* and *rBias* were calculated for *f*AUC_0–24h_/MIC and *f*C_min_ predictions based on information from one, two and three sampling occasions. Both the accuracy and precision in predictions of *f*AUC_0–24h_/MIC (*rBias*: −5.0%, −2.1%, and −1.8%; *rRMSE*: 19.3%, 12.4%, and 8.1% for one, two, and three occasions) and *f*C_min_ (*rBias*: −8.9%, −2.7%, and −2.1%; *rRMSE*: 44.8%, 30.5%, and 20.2% for one, two and three occasions) improved when additional information was added.

**Figure 5:**
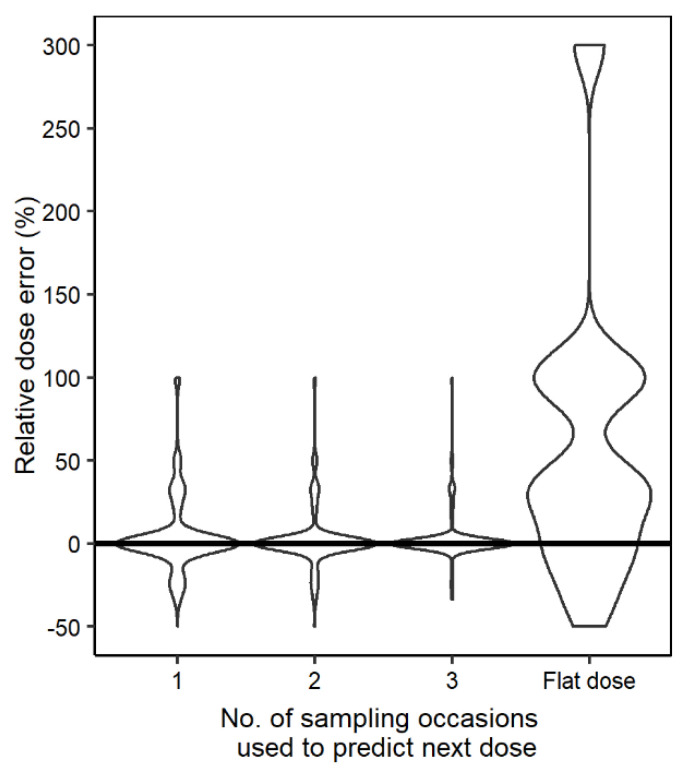
Violin plot showing the dose prediction error comparing the true dose to the individualized dose derived based on information from one, two, and three sampling occasions, as well as flat dosing of 600 mg QD for comparison.

The relative dose prediction error decreased with increasing information used to obtain individual parameters from sparse sampling (Figure 5), indicating that a higher percentage of simulated individuals received a dose closer to the true dose.

## 4. Discussion

An MIPD workflow, using the here developed population PK model, was established, enabling safe and efficacious dosing on an individual level.

Several studies have shown that linezolid clearance decreases with increasing doses. This phenomenon has previously been described with either Michaelis–Menten elimination kinetics [36,37] or concentration- and time-dependent auto-inhibition of elimination [22,25,26]. In this work, Michaelis–Menten elimination kinetics was not supported by the data; however, the inclusion of a concentration- and time-dependent auto-inhibition of elimination described the data well, and its incorporation is crucial to be able to use the population PK model for MIPD before steady state is reached. The mechanism behind the auto-inhibition of linezolid elimination is not fully known yet. It is suggested that for the metabolite’s hydroxyethyl glycine formation, reduced nicotinamide adenine dinucleotide phosphate (NADPH) is needed [22,38]. As linezolid inhibits cytochrome *c*-oxidase activity, it interrupts the synthesis of adenosine triphosphate (ATP), which is needed for nicotinamide adenine dinucleotide phosphate (NADP) reduction to NADPH [22]. Lack of NADPH results in decreased hydroxylinezolid formation, and thus, linezolid elimination is inhibited.

In this work, both statistical and clinical significance were considered when incorporating covariates in the model. Three covariates fulfilled both criteria, HIV co-infection on *CL/F*, sex on *k_a_*_,_ and coadministration of P-gp inhibitors on *MTT* (Table 2). Typical *CL/F* was 43% higher in patients with HIV co-infection, and the effect of the covariate on the apparent clearance could be explained by changes in kidney function or concomitant medication (Table 2). Typical *k_a_* was 95% higher in females, possibly due to biological differences between men and women, such as differences in gastric pH, gastric fluid flow, intestinal motility, and gastric emptying [39]. Typical *MTT* was 96% higher in patients who received P-gp inhibitors. The imprecision in the estimated effect of HIV on *CL/F* was high, with a 90% confidence interval of 7–90% (Table 2), which is probably due to the low number of HIV patients in the study population (n = 5/70). The covariate was kept in the model since clearance is a parameter that can greatly influence plasma concentration. However, the effect of HIV on *CL/F* estimated here should be interpreted with caution and studied further. Allometric scaling, besides improving the model fit, was implemented in order to be able to apply the population PK model to a pediatric population.

Linezolid has high efficacy for the treatment of MDR- and XDR-TB and a low resistance development rate but is one of the most frequently reported anti-TB drugs to cause severe adverse events, which might require early termination of treatment [40]. A recent clinical trial investigating long-term linezolid treatment showed that toxicity was high, with 81% of the patients suffering from peripheral neuropathy and 48% from myelosuppression [8]. According to our simulations, a flat dosing regimen of 600 mg QD led to efficacious and safe exposures in 67.2% of the simulated patients, which is comparable to previous findings [5,6,41]. While some work suggests that 600 mg BID is needed to reach efficacy [42], other studies are in accordance with the here described findings. In the work by Millard et al. [6], the majority of simulated patients reached the safety target following doses of 300 mg QD, 300 mg BID, and 600 mg QD, but almost all subjects were above the safety threshold with a dose of 600 mg BID. Alghamdi et al. [41] also suggested that 600 mg QD is preferred in most patients with respect to safety. Furthermore, recent results from the ZeNix trial presented in Berlin 2021 [43] highlight that cure is achievable with 600 mg QD for 6 months, leading to less frequent adverse events compared to 1200 mg QD (38% vs. 24% for peripheral neuropathy and 22% vs. 2% for anemia) [43].

Besides studying the use of lower doses to increase safety, as in the ZeNix trial (NCT03086486), individualizing a patient’s dose based on individual characteristics and drug exposure with MIPD can be used as a tool to increase efficacy and safety [13,14,15,16]. MIPD provides individual dose suggestions, which is different from classical TDM, where the achieved PK exposure after a dose is compared to a target without a precise dose suggestion apart from the information that a dose is too high or too low. The here proposed MIPD approach (Figure 3) ensures efficacy and safety on an individual level and can be applied at any time during treatment. Simulations of 1000 virtual patients showed that by using the MIPD approach, 88.2% reached efficacious and safe linezolid concentrations with an individualized dose obtained using information from three sampling occasions. After three sampling occasions, 6.9% of all simulated patients would get an efficacious but not safe dose (17.6% following a flat dose of 600 mg QD), 4.3% would get a safe but not efficacious dose (versus 14% for 600 mg QD flat dosing), and for 0.3% of the simulated patients, the dose would be considered neither safe nor efficacious (versus 1.2% for 600 mg QD flat dosing) (see Figure 4). Using information from three occasions resulted in 88.2% of simulated patients reaching both the efficacy and safety target (improvement of 6.7% compared to using information from two occasions), thus highlighting the importance of the third occasion (Figure 4). In all simulated patients, a QD dosing strategy was superior compared to BID due to the fact that the *f*C_min_ is lower following QD dosing. The superiority of a QD dosing strategy over BID has been shown previously [5,6,41] and could be advantageous to increase patient adherence.

There are some limitations to this work. Firstly, since retrospective TDM data from routine clinical care was analyzed and because patients were in their intensive phase of TB infection, selection bias might have been introduced. In addition, it was only possible to derive the dose prediction error for the total daily dose; thus, there was no distinction between QD and BID dosing. However, since a QD strategy was superior to BID in all simulated patients, it was not necessary here to compare the dose prediction error between QD versus BID strategies. Furthermore, the parameters describing the auto-inhibition of elimination were not identifiable in this work as the majority of samples were taken at steady state, and thus the parameters related to elimination auto-inhibition had to be fixed to values obtained from earlier studies [25]. In this work, efficacy and safety targets from earlier defined PKPD indices were used for dose selection. A simulation study by Kristoffersson et al. [44] showed that the use of longitudinal PKPD models can be more appropriate in certain situations than using PKPD indices. PKPD indices can further be influenced by uncertainty in the obtained MIC value, both due to intra- and inter-laboratory variability [45,46]. Since the MIC is determined in two-fold dilution steps, an error may lead to a substantial change in the PKPD index and, subsequently, dose selection. These findings indicate that MIPD based on a PKPD model might be more appropriate than based on PKPD indices, which should be investigated in further studies. Furthermore, the safety target has only been evaluated in one study with 38 participants [7], and the authors merely explored *f*C_min_ as a potential driver for toxicity. Other PK parameters such as AUC or C_max_ were not explored, and there is, therefore, a need to derive an updated individualized safety target based on PK data combined with clinical safety outcome data. Lastly, the developed MIPD approach should be validated in the clinic.

## 5. Conclusions

In conclusion, a linezolid population PK model for MDR- and XDR-TB patients was successfully developed. This work presents an MIPD workflow for linezolid, which can be used on any day of treatment, proposes to use three sparse sampling occasions to derive the individualized dose, and suggests that an individualized dose would be beneficial from an efficacy and safety perspective compared to a flat dose of 600 mg QD.

## Figures and Tables

**Table 1:** Demographics and covariates for patients included in the data set used for population pharmacokinetic model building.

Parameter	Unit	All Patients
N		70
Mean weight (range)	kg	61.2 (35.3–88.9)
Mean height (range)	m	1.70 (1.50–1.93)
Mean creatinine clearance (range)	mL/min	116.1 (40.7–150.0) ^a^
Mean age (range)	years	32 (15–70)
Mean body mass index (range)	kg/m^2^	21.2 (15.5–32.6)
No. of male sex	n (%)	38 (54.5)
No. with HIV	n (%)	5 (7.1)
No. with diabetes	n (%)	9 (12.9)
No. smoking	n (%)	26 (37.1)
No. alcohol abuse	n (%)	6 (8.6)
No. pregnancy	n (%)	3 (4.3)
No. from indicated WHO region	n (%)	
African region		10 (14.3)
Region of the Americas		2 (2.9)
Southeast Asia region		6 (8.6)
European region Eastern		27 (38.6)
Mediterranean region		15 (21.4)
Western Pacific region		10 (14.3)

^a^ Calculated using the Cockcroft-Gault equation [18], using lean body weight instead of regular body weight for patients with BMI higher than 25 and with creatinine clearance truncated at 150 mL/min (13 patients had a calculated creatinine clearance above 150 mL/min). Age, bodyweight, body mass index, and creatinine plasma concentration were registered on the day of admission. WHO region–region based on World Health Organization (WHO) region classification describing origin of birth; ART–antiretroviral therapy; alcohol–alcohol abuse characterized by more than 1 or 2 glasses of alcohol/day and less than 2 days/week with no alcohol; n–number of patients.

**Table 2:** Parameter estimates from the final linezolid population pharmacokinetic model.

Parameter	Description	Estimate	90% CI ^c^	RSE% ^d^
*CL/F* (L/h/70 kg)	Apparent clearance (uninhibited)	6.3	5.6–7.0	6.4
*V_d_/F* (L/70 kg)	Apparent volume of distribution	50.6	48.5–53.1	3.1
*ka* (h^−1^)	Absorption rate constant	1.8	1.5–2.1	13.8
*MTT* (h)	Mean transit time	0.53	0.44–0.61	10.6
*k_IC_* (h^−1^)	Rate constant into the inhibition compartment	0.0005 FIX ^e^	-	-
*IC_50_* (mg/L)	Concentration in the inhibition compartment yielding half of clearance inhibition	0.38 FIX ^e^	-	-
*RCLF*	Remaining clearance fraction uninhibited	0.798	0.69–0.92	11.3
**Covariates**				
HIV co-infection on *CL/F*	Effect of HIV co-infection on *CL/F*	0.43	0.07–0.90	122.0
Sex on *k_a_*	Effect of sex on *k_a_*	0.95	0.78–1.10	14.0
P-gp inhibitor on *MTT*	Effect of P-gp inhibitor on *MTT*	0.96	0.84–1.09	9.0
**Inter-individual variability**				
IIV*_CL/F_* (%CV) ^a^	Inter-individual variability in apparent clearance (uninhibited)	0.26	0.21–0.31	13.0
IIV*_MTT_* (%CV) ^a^	Inter-individual variability in mean transit time	0.62	0.40–0.80	19.9
**Inter-occasion variability**				
IOV*_CL/F_* (%CV) ^b^	Inter-occasion variability in apparent clearance (uninhibited)	0.27	0.23–0.30	9.0
IOV*_V/F_* (%CV) ^b^	Inter-occasion variability in apparent volume of distribution	0.26	0.23–0.30	8.5
IOV*_ka_* (%CV) ^b^	Inter-occasion variability in absorption rate constant	0.93	0.71–1.16	15.2
IOV*_MTT_* (%CV) ^b^	Inter-occasion variability in mean transit time	0.69	0.53–0.85	13.4
**Residual variability**				
Proportional error (%)	Proportional residual error	0.054	0.045–0.065	12.5
Additive error (mg/L)	Additive residual error	0.53	0.483–0.570	7.0

^a^ Inter-individual variability expressed as the standard deviation and in % of the parameter estimate. ^b^ Inter-occasion variability expressed as the standard deviation and in % of the parameter estimate. ^c^ 90% CI is the 90% percentile confidence interval obtained from a sampling importance resampling (SIR) procedure. ^d^ Standard errors expressed as relative standard errors (standard errors for omegas relative to their variance estimates). ^e^ Values obtained by a publication by Keel et al. [25]. IIV, inter-individual variability; IOV, inter-occasion variability; RSE, residual standard error.

## Data Availability

The data presented in this study are available on request from the corresponding author. The data are not publicly available due to privacy reasons, as this is sensitive personal patient data.

## Supplementary Materials

The following supporting information can be downloaded at: https://www.mdpi.com/article/10.3390/pharmaceutics14040753/s1, Text S1 NONMEM model code for the final population pharmacokinetic model; Figure S1 Goodness-of-fit plots for the final population pharmacokinetic model; Figure S2 Prediction corrected visual predictive checks (pcVPC) for the final linezolid population pharmacokinetic model stratified on covariates; Figure S3 Sankey plot showing individual dose adjustments for three consecutive PK sampling occasions, each one week apart; Table S1 Summary of the different regimens included in the analysis dataset; Table S2 Covariate relationships evaluated in the population pharmacokinetic analysis.
