# Supplementary material for: Model-Informed Precision Dosing of Linezolid in Patients with Drug-Resistant Tuberculosis
Source: Pharmaceutics. 2022 Mar 30;14(4):753. doi: 10.3390/pharmaceutics14040753 (PMC9032906; doi:10.3390/pharmaceutics14040753)
Supplement: Supplementary file 1 [file pharmaceutics-14-00753-s001.zip › pharmaceutics-1617847-supplementary.pdf]

Supplementary materials

Model-Informed Precision Dosing of Linezolid in Patients with Drug-Resistant Tuberculosis

Laurynas Mockeliunas, Lina Keutzer, Marieke G.G. Sturkenboom, Mathieu S. Bolhuis, Lotte M.G. Hulskotte, Onno W. Akkerman and Ulrika S.H. Simonsson

Email: ulrika.Simonsson@farmbio.uu.se

Table S1. Summary of the different regimens included in the analysis dataset

Regimen	Number of PK sampling occasions (total = 155)*	Proportion of total PK sampling occasions
150 mg QD	7	0.045
200 mg QD	5	0.032
200 mg BID	8	0.052
300 mg QD	33	0.213
300 mg BID	53	0.342
400 mg QD	1	0.006
600 mg QD	34	0.219
600 mg BID	14	0.090

*Number of PK sampling occasions represents the total number of occasions as some patients had more than one PK sampling occasion; BID – dosing every 12 hours; QD – dosing every 24 hours

Table S2. Covariate relationships evaluated in the population pharmacokinetic analysis

Parameter	Type of covariate	Covariate
CL/F (L/h/70 kg)	time-constant, continuous covariates	age, creatinine clearance[a]
	time-constant, categorical covariates	alcohol abuse, sex, diabetes, HIV co-infection, pre-emptive use of erythropoietin, smoking, origin of birth (WHO region)
	time-varying, categorical covariates	P-gp inhibitors, P-gp inducers, CYP3A4 inhibitors, CYP3A4 inducers
V/F (L/70 kg)	time-constant, categorical covariates	alcohol abuse, sex, diabetes, HIV co-infection, pre-emptive use of erythropoietin, smoking, origin of birth (WHO region)
k_a (h^{-1})	time-constant, categorical covariates	alcohol abuse, sex, diabetes, HIV co-infection, smoking
	time-varying, categorical covariates	P-gp inhibitors, P-gp inducers
MTT (h)	time-constant, categorical covariates	alcohol abuse, sex, diabetes, HIV co-infection, smoking
	time-varying, categorical covariates	P-gp inhibitors, P-gp inducers

[a]Calculated using the Cockcroft-Gault equation [1], using lean body weight instead of regular body weight for patients with BMI higher than 25, and with creatinine clearance truncated at 150 mL/min (13 patients had a creatinine clearance above 150 mL/min).

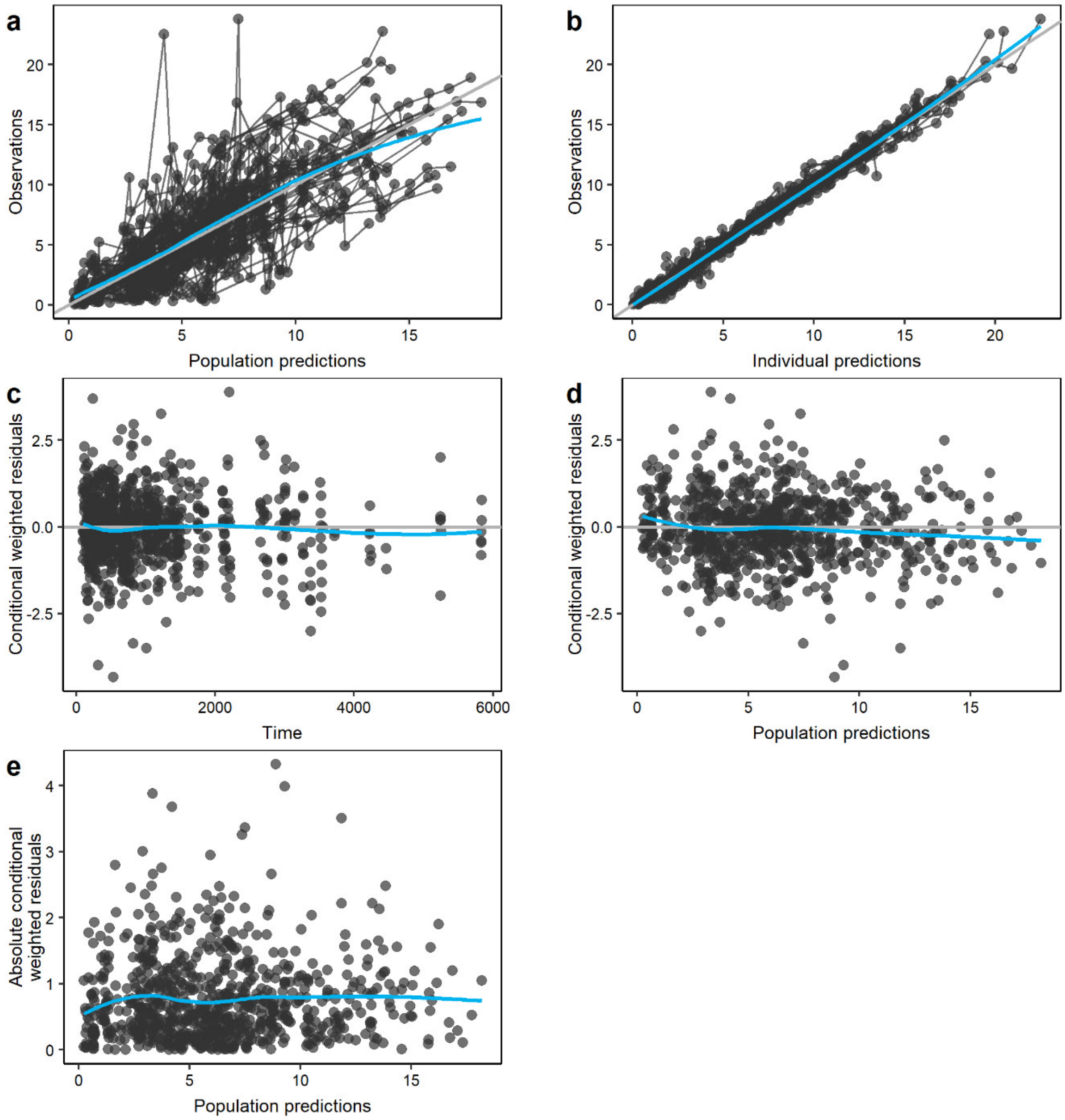

Figure S1. Goodness-of-fit plots for the final population pharmacokinetic model. Panel a–Population predictions vs. Observations, b–Individual predictions vs. Observations; c–Conditional weighted residuals vs. Time, d–Conditional weighted residuals vs. Population predictions, e–Absolute conditional individual weighted residuals vs. Population predictions.

In panels a and b, the grey line represents the line of identity, while the blue line is a smoothed trendline through data. The trendless flat blue line going through zero abscissa in panel c indicates that the structural model describes the data well; the trendless flat blue line in panel d indicates that there are no model misspecification present; the trendless flat blue line in panel e shows that the selected error model is appropriate.

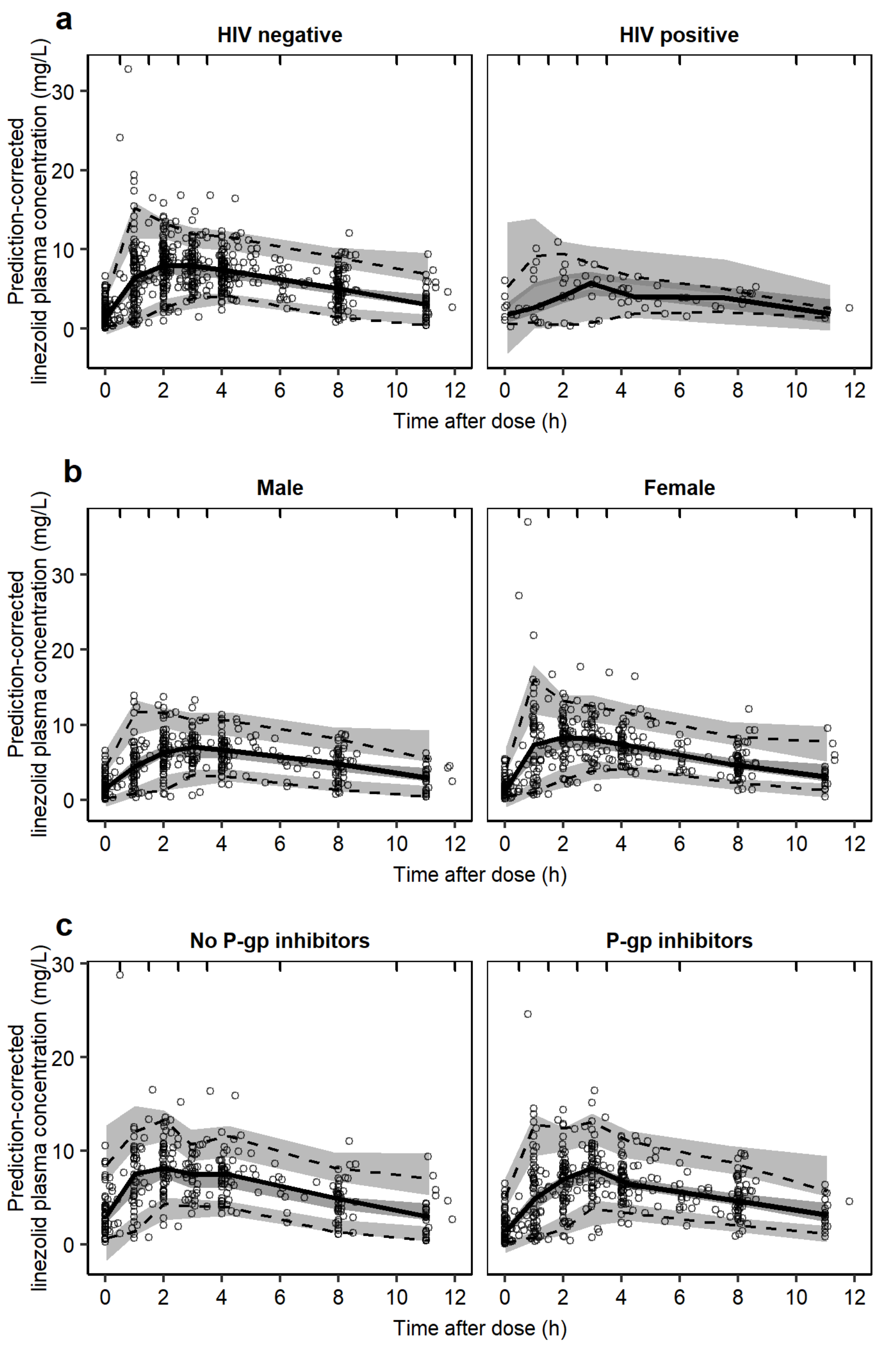

Figure S2. Prediction corrected visual predictive checks (pcVPC) for the final linezolid population pharmacokinetic model stratified on covariates. The solid and dashed lines are the median, 2.5th and 97.5th percentiles of the observed data, respectively. The shaded areas (top to bottom) are the 95% confidence intervals of the 97.5th (light grey), median (grey) and 2.5th (light grey) percentiles of the simulated data based on 1000 simulations. Open circles are prediction-corrected observation points. Panel a shows a pcVPC stratified on the effect of HIV co-infection on *CL/F*, while panel b shows a pcVPC stratified on the effect of sex on k_a, and panel c shows a pcVPC stratified on the effect of co-administration of P-gp inhibitors on *MTT*.

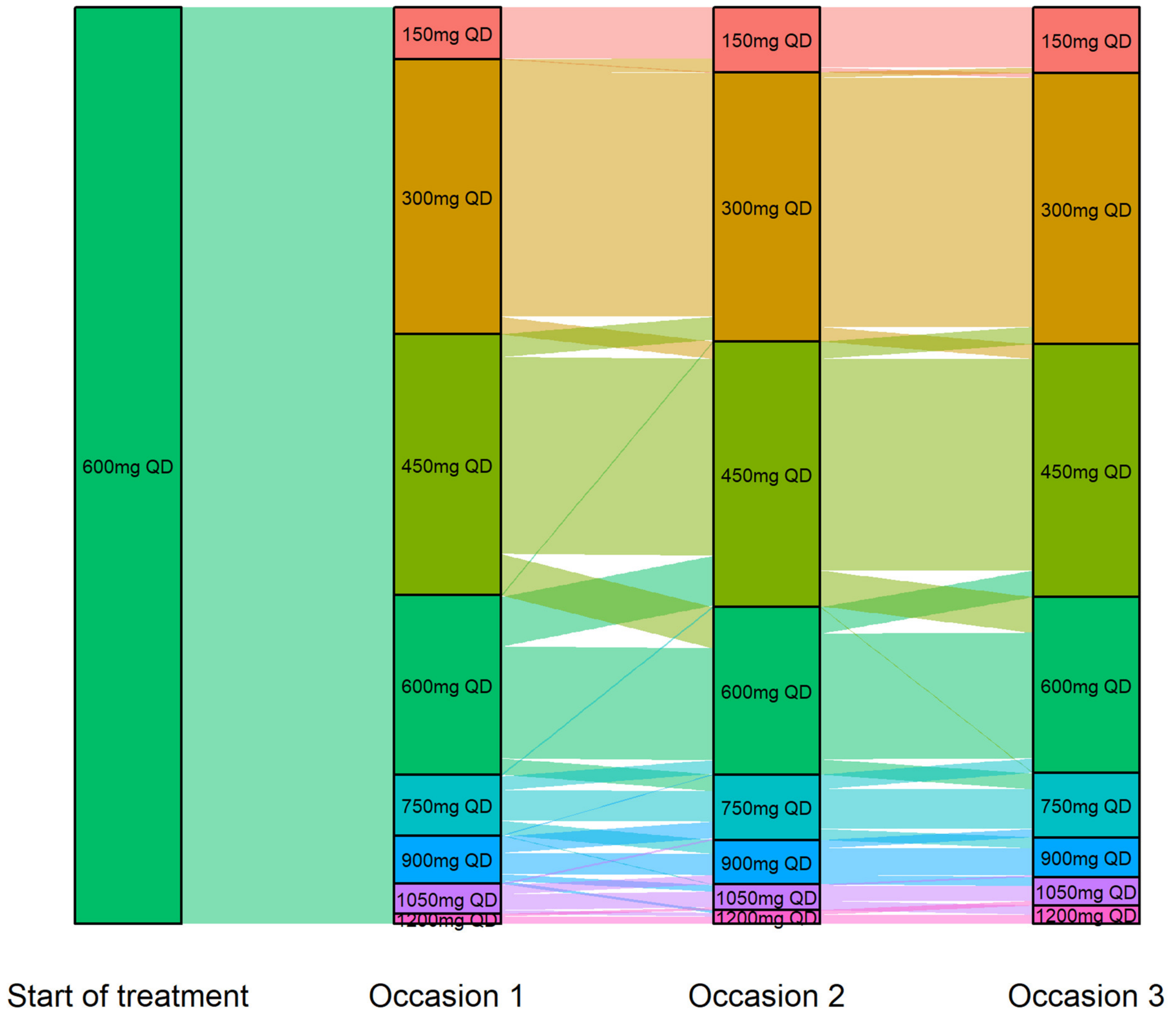

Figure S3. Sankey plot showing individual dose adjustments for three consecutive PK sampling occasions, each one week apart. The size of the boxes represents the proportion of patients falling within the respective category. The lines show the proportion of patients transitioning to another category, here dosing regimen.

QD, once daily; BID, twice daily.

Text S1. NONMEM model code for the final population pharmacokinetic model

```
$SIZES LVR=50

$SIZES LNP4=60000

$PROBLEM Final Linezolid POPPK model with inhibition compartment

$INPUT ID AMT II DV OCC LOQ TAD TIME ADDL DRUG DATASET DOSE
INTERVAL STRAT PLOT CMT=DROP FLAG EVID DAYS BWAD AGE SEX WHO
HIV DIABETES SMOKING ALCOHOL PREEPO CLCR PGP_IND PGP_INH
CYP_INH CYP_IND

; ID - patient identifier

; AMT – linezolid amount administered (mg)

; II - dosing interval, needed for ADDL

; DV - dependent variable, plasma concentrations

; OCC - sampling occasion

; LOQ - indicator, if value was below LLOQ, if yes, DV was set to LLOQ/2

; TAD - time after dose

; TIME - time from start of enrolment

; ADDL - additional daily doses, required

; DRUG - indicator if Zyvoxid or generic was administered

; DATASET - indicator which dataset the data came from

; DOSE - AMT for plotting purposes

; INTERVAL - II for plotting purposes

; STRAT - Variable for plotting purposes, AMT+II

; PLOT - variable for plotting purposes, OCC+AMT+II

; CMT - compartment

; FLAG - variable for AUC calculation

; EVID - event ID

; DAYS - time in days
```

```
; BWAD - bodyweight at admission

; AGE – age in years

; SEX - sex

; WHO - WHO region that the patient was born in

; HIV - HIV status

; DIABETES - diabetes status

; SMOKING - smoking status

; ALCOHOL - alcohol abuse, more than 1 or 2 glasses of alcohol/day and less than 2 days/week no alcohol (before TB treatment)

; PREEPO - Pre-emptive use of erythropoietin

; CLCR - creatinine clearance, calculated using Cockcroft-Gault formula

; PGP_IND - administration of P-gp inducers

; PGP_INH - administration of P-gp inhibitors

; CYP_INH - administration of CYP3A4 inhibitors

; CYP_IND - administration of CYP3A4 inducers

$DATA       ../data.csv IGNORE=@

$SUBROUTINE ADVAN13 TRANS1 TOL=10

$MODEL NCOMP=8 COMP=(DEPOT,DEFDOSE) COMP=(CENTRAL,DEFOBS) COMP=(TRANSIT2) COMP=(TRANSIT3) COMP=(TRANSIT4) COMP=(TRANSIT5) COMP=(ABS) COMP=(INH)

;; The first compartment is the first transit compartment

$PK

;;;;;CL covariates relationships;;;;;

;;; CLHIV-DEFINITION START

IF(HIV.EQ.0) CLHIV=1; Most common case, indicator variable is 1

IF(HIV.EQ.1) CLHIV=(1+THETA(10))

;;; CLHIV-DEFINITION END
```

```
;;; CL-RELATION START
CLCOV=CLHIV
;;; CL-RELATION END
;;;;;KA covariates relationships;;;;;
;;; KASEX-DEFINITION START
IF(SEX.EQ.0) KASEX=1
IF(SEX.EQ.1) KASEX=(1+THETA(11))
;;; KASEX-DEFINITION END

;;; KA-RELATION START
KACOV=KASEX
;;; KA-RELATION END
;;;;;MTT  covariates relationships;;;;;
;;; MTTPGP_INH-DEFINITION START
IF(PGP_INH.EQ.0) MTTPGP_INH=1
IF(PGP_INH.EQ.1) MTTPGP_INH=(1+THETA(12))
;;; MTTPGP_INH-DEFINITION END
;;; MTT-RELATION START
MTTCOV=MTTPGP_INH
;;; MTT-RELATION END
TVCL    = THETA(1)*(BWAD/70)**0.75   ;  apparent clearance (CL)
TVCL    = CLCOV*TVCL
TVV     = THETA(2)*(BWAD/70) ; apparent volume of distribution (V)
TVKA    = THETA(3)        ; absorption rate constant (ka)
TVKA    = KACOV*TVKA
TVMTT   = THETA(6)        ; mean transit time (MTT)
```

```
TVMTT   = MTTCOV*TVMTT

TVKIC   = THETA(7)        ; rate constant into the inhibition compartment

TVRCLF  = THETA(8)        ; remaining clearance fraction uninhibited

TVIC50  = THETA(9)        ; concentration in the inhibition compartment yielding half of CL inhibition

IOV_CL   = 0              ; IOV on apparent clearance

IF(OCC.EQ.1) IOV_CL = ETA(4)

IF(OCC.EQ.2) IOV_CL = ETA(5)

IF(OCC.EQ.3) IOV_CL = ETA(6)

IF(OCC.EQ.4) IOV_CL = ETA(7)

IF(OCC.EQ.5) IOV_CL = ETA(8)

IF(OCC.EQ.6) IOV_CL = ETA(9)

IF(OCC.EQ.7) IOV_CL = ETA(10)


IOV_V   = 0          ; IOV on apparent volume of distribution

IF(OCC.EQ.1) IOV_V = ETA(11)

IF(OCC.EQ.2) IOV_V = ETA(12)

IF(OCC.EQ.3) IOV_V = ETA(13)

IF(OCC.EQ.4) IOV_V = ETA(14)

IF(OCC.EQ.5) IOV_V = ETA(15)

IF(OCC.EQ.6) IOV_V = ETA(16)

IF(OCC.EQ.7) IOV_V = ETA(17)


IOV_KA   = 0        ; IOV on absorption rate constant

IF(OCC.EQ.1) IOV_KA = ETA(18)

IF(OCC.EQ.2) IOV_KA = ETA(19)
```

```
IF(OCC.EQ.3) IOV_KA = ETA(20)
IF(OCC.EQ.4) IOV_KA = ETA(21)
IF(OCC.EQ.5) IOV_KA = ETA(22)
IF(OCC.EQ.6) IOV_KA = ETA(23)
IF(OCC.EQ.7) IOV_KA = ETA(24)

IOV_MTT   = 0       ; IOV on mean transit time
IF(OCC.EQ.1) IOV_MTT = ETA(25)
IF(OCC.EQ.2) IOV_MTT = ETA(26)
IF(OCC.EQ.3) IOV_MTT = ETA(27)
IF(OCC.EQ.4) IOV_MTT = ETA(28)
IF(OCC.EQ.5) IOV_MTT = ETA(29)
IF(OCC.EQ.6) IOV_MTT = ETA(30)
IF(OCC.EQ.7) IOV_MTT = ETA(31)
CL      = TVCL*EXP(ETA(1) + IOV_CL)
V       = TVV*EXP(IOV_V)
KA      = TVKA*EXP(IOV_KA)
K20     = CL/V ; elimination rate constant
MTT     = TVMTT*EXP(ETA(2) + IOV_MTT)
NN      = 5    ; number of transit compartments (hard-coded)

KIC     = TVKIC
RCLF    = TVRCLF
IC50    = TVIC50
KTR     = (NN+1)/MTT
K13     = KTR
```

```
K34    = KTR
K45    = KTR
K56    = KTR
K67    = KTR
K72    = KA

$DES
CP     = A(2)/V
DADT(1) = -KTR*A(1)
DADT(2) = KA*A(7)- K20*A(2)*(RCLF+(1-RCLF)*(1-A(8)/(IC50+A(8))) ;central compartment
DADT(3) = KTR*A(1)- KTR*A(3)
DADT(4) = KTR*A(3)- KTR*A(4)
DADT(5) = KTR*A(4)- KTR*A(5)
DADT(6) = KTR*A(5)- KTR*A(6)
DADT(7) = KTR*A(6)- KA*A(7)    ; absorption compartment
DADT(8) = KIC*(CP - A(8))      ; inhibition compartment

$ERROR
IPRED = A(2)/V     ; combined residual error
IRES = DV - IPRED
W = SQRT((THETA(4)*IPRED)**2+THETA(5)**2)
IWRES = IRES/W
Y = IPRED+W*EPS(1)
A1  = A(1)        ; transit compartment
A2  = A(2)        ; central compartment
```

```
A3  = A(3)         ; transit compartment
A4  = A(4)         ; transit compartment
A5  = A(5)         ; transit compartment
A6  = A(6)         ; transit compartment
A7  = A(7)         ; absorption compartment
A8  = A(8)         ; inhibition compartment

$THETA
 (0,6.10679)       ; CL
 (0,53.9716)       ; V
 (0,2.50423)       ; KA
 (0,0.268449)      ; PROP ERROR
 (0,0.578366)      ; ADD ERROR
 (0,0.718694)      ; MTT
 0.0005 FIX        ; KIC
 (0,0.894458,1)    ; RCLF
 0.38 FIX          ; IC50
 (-1,0.571775,5)   ; CLHIV1
 (-1,0.988896,5)   ; KASEX1
 (-1,0.988896,5)   ; MTTPGP_INH1
$OMEGA
 0.0739769         ; IIV_CL
 0.402776          ; IIV_MTT

$OMEGA BLOCK(1) 2.48E-02     ; IOV_CL
$OMEGA BLOCK(1) SAME         ; IOV_CL
```

```
$OMEGA BLOCK(1) SAME        ; IOV_CL
$OMEGA BLOCK(1) SAME        ; IOV_CL
$OMEGA BLOCK(1) SAME        ; IOV_CL
$OMEGA BLOCK(1) SAME        ; IOV_CL
$OMEGA BLOCK(1) SAME        ; IOV_CL

$OMEGA BLOCK(1) 2.48E-02    ; IOV_V
$OMEGA BLOCK(1) SAME        ; IOV_V
$OMEGA BLOCK(1) SAME        ; IOV_V
$OMEGA BLOCK(1) SAME        ; IOV_V
$OMEGA BLOCK(1) SAME        ; IOV_V
$OMEGA BLOCK(1) SAME        ; IOV_V
$OMEGA BLOCK(1) SAME        ; IOV_V
$OMEGA BLOCK(1) 2.48E-02    ; IOV_KA
$OMEGA BLOCK(1) SAME        ; IOV_KA
$OMEGA BLOCK(1) SAME        ; IOV_KA
$OMEGA BLOCK(1) SAME        ; IOV_KA
$OMEGA BLOCK(1) SAME        ; IOV_KA
$OMEGA BLOCK(1) SAME        ; IOV_KA
$OMEGA BLOCK(1) SAME        ; IOV_KA
$OMEGA BLOCK(1) 2.48E-02    ; IOV_MTT
$OMEGA BLOCK(1) SAME        ; IOV_MTT
$OMEGA BLOCK(1) SAME        ; IOV_MTT
$OMEGA BLOCK(1) SAME        ; IOV_MTT
$OMEGA BLOCK(1) SAME        ; IOV_MTT
$OMEGA BLOCK(1) SAME        ; IOV_MTT
```

```
$OMEGA BLOCK(1) SAME        ; IOV_MTT

$SIGMA  1  FIX            ; RESIDUAL ERROR

$ESTIMATION METHOD=1 INTER MAXEVAL=9999 NSIG=3 SIGL=9 PRINT=3
NOABORT

$COVARIANCE PRINT=E MATRIX=R

$TABLE  ID IPRED IWRES CWRES NPDE DV TIME TAD OCC EVID INTERVAL
STRAT PLOT            NOPRINT ONEHEADER FILE=sdtab1

$TABLE  ID DOSE DRUG DATASET SEX WHO HIV DIABETES SMOKING
ALCOHOL                    NOPRINT ONEHEADER FILE=catab1

$TABLE  ID BWAD AGE PREEPO CLCR  NOPRINT ONEHEADER FILE=cotab1

$TABLE  ID CL V KA MTT RCLF IC50 KIC W ETA1 ETA2

                        NOPRINT ONEHEADER FILE=patab1
```

References

[1] Cockcroft, D.W.; Gault, M.H. Prediction of creatinine clearance from serum creatinine. *Nephron* **1976**, 16:31–41. https://doi.org/10.1159/000180580.
